# Infantile epileptic spasms syndrome: a cohort study of 88 children

**DOI:** 10.1186/s13052-023-01563-z

**Published:** 2023-12-01

**Authors:** Li-Hong Ren, Jing Zhang, Si-Xiu Li, Ping Liu, Hui Chen, Wenguang Hu

**Affiliations:** Department of Pediatric Neurology, School of Medicine, Chengdu Women’s and Children’s Central Hospital, University of Electronic Science and Technology of China, No. 1617, Riyue Aveneue, Chengdu, 611731 China

**Keywords:** Infantile epileptic spasms syndrome, Etiology, Risk factors, Etiology-specific

## Abstract

**Background:**

This study aimed to investigate and analyze the risk factors for non-etiology-specific infantile spasms (IS) and unrelieved clinical symptoms after treatment.

**Methods:**

Eighty-eight children with IS who were treated at our hospital from March 2018 to December 2021 were included in the study. The children were divided into etiology-specific (n = 46) and nonetiology-specific (n = 42) groups, based on the diagnostic results, and remission (n = 45) and nonremission (n = 43) groups, based on clinical outcomes after treatment. The clinical data from patients in the etiology-specific and nonetiology-specific groups and the remission and nonremission groups were compared. Risk factors for non-etiology-specific IS were identified using logistic regression analysis.

**Results:**

Gender, family history, birth status, and metabolic abnormalities were significantly different between the etiology-specific and non-etiology-specific groups. Gender and metabolic abnormalities were risk factors for nonetiology-specific IS. Family history, birth status, metabolic abnormalities, and brain magnetic resonance imaging were significantly different between the remission and nonremission groups, and different etiologies were risk factors for unrelieved symptoms after treatment.

**Conclusion:**

The occurrence of nonetiology-specific IS is associated with gender and metabolic abnormalities in children. After medication, unrelieved IS symptoms are associated with etiologies.

## Background

Infantile spasms (IS), also known as West syndrome and infantile epileptic spasms syndrome, is a serious epileptic encephalopathy of early childhood with an incidence of about 1 case per 2000 to 4000 live births. IS is characterized by unique seizures, including flexor, extensor, or mixed spasms, and transient contractions usually occur in the neck, trunk, and limb muscles [1–4]. Most cases of IS occur at 3 to 7 months of age. According to the International League Against Epilepsy classification [5], IS can be divided into two major categories: (1) self-limited epilepsy syndromes in which spontaneous remission is likely to occur and (2) developmental and epileptic encephalopathies (DEEs), which are accompanied by developmental impairment related to both the underlying etiology independent of the epileptiform activity and the epileptic encephalopathy. IS syndrome is one of the most devastating neurological diseases for infants and young children due to the difficult antiepileptic treatment and serious intellectual prognosis [6, 7].

Although the proportion of etiology-specific IS is expanding, the majority of patients are still diagnosed with non-etiology-specific IS [8]. Etiology-specific epilepsy syndrome is defined as IS with specific genetic, structural, metabolic, immune, or infectious etiologies, and consistent electroclinical features [9]. Most etiology-specific syndromes that begin in the neonatal or infantile period are DEEs [5]. Currently, more than 200 etiologies for IS have been identified [10]. Perinatal injury accounts for more than 60% of IS patients; structural etiologies include developmental malformations of the brain and cortex such as focal cortical dysplasia, polymicrogyria, and tuberous sclerosis [11]. IS with etiological heterogeneity is difficult to treat [12].

Some studies recommend that high-quality magnetic resonance imaging (MRI) be performed in all IS patients, and metabolic/genetic tests should be performed if etiologies are not identified [13]. With advances in genetic and imaging techniques, more IS etiologies have been identified; however, only two-thirds of etiological events in children are identified [14]. Therefore, a cohort study of 88 infants admitted to our hospital was conducted to investigate the etiology of IS and the risk factors for non-etiology-specific spasms and unrelieved symptoms after medication in children with IS.

## Materials and methods

### Clinical data

Eighty-eight children with IS diagnosed in the Chengdu Women’s and Children’s Central Hospital from March 2018 to December 2021 were included in the study. Based on the diagnostic results, patients were divided into etiology-specific (n = 46) and non-etiology-specific (n = 42) groups. Patients were also classified into remission (n = 45) and nonremission (n = 43) groups based on clinical outcomes after treatment. Symptom remission was defined as a sustained reduction of seizure frequencies ≥ 50% after treatment [15].

Patient data, including gender, age, family history, birth status (absence or presence of birth abnormalities such as prematurity and/or asphyxia), metabolic status (normal or abnormal detected during screening for neonatal genetic and metabolic diseases), age and duration of onset (time from the onset to the present day), were recorded. All children’s families signed informed consent. This study was approved by the Ethics Committee of Chengdu Women’s and Children’s Central Hospital (2022-018).

Inclusion criteria: etiology was determined using brain MRI, metabolic examination, and genetic diagnosis. Typical electroencephalogram hypoarrhythmia (HY) and epileptic spasm recorded by video electroencephalogram were present. Exclusion criteria: Data were incomplete. Outcome measures: risk factors of infantile spasms in children.

### Magnetic resonance imaging (MRI) examination

MRI examination was performed using a 1.5- or 3.0-Tesla system. Fluid attenuated inversion recovery image acquisition parameters consisted of field-of-view (200–240 mm), repetition time (TR: 5000–13,000 ms), echo time (TE: 91–169 ms), slice thickness (ST: 1.0–4.0 mm), and the number of excitations (NEX: 1–2). T1-weighted image parameters included TR (8.3–2250 ms), TE (3.2–15 ms), ST (1.4–5.0 mm), and NEX (1–2).

### Statistical analysis

Statistical analyses were performed with SPSS 26.0. Measurement data were expressed as means ± standard deviations, and groups were compared using t tests. Enumeration data were expressed as n or %, and groups were compared using χ^2^ tests. Logistic regression analysis was applied to predict risk factors. *P* < 0.05 was considered statistically significant.

## Results

### Baseline characteristics of children in the etiology-specific and non-etiology-specific groups

The 88 children (age: 260.58 ± 147.08 days; the mean hospital stay: 28.20 ± 10.22 days) enrolled in the study included 44 children with family histories of epilepsy, 37 cases of normal delivery, 12 cases of premature delivery, 19 cases of asphyxia, and 20 cases with other birth abnormalities. Forty-six children had specific etiologies (etiology-specific group) and 42 children lacked etiologies (non-etiology-specific group). The etiology-specific and non-etiology-specific groups were compared to identify differences in clinical data. No differences were detected between the two groups in age (255.93 ± 173.93 days vs. 254.74 ± 119.03 days), age of onset (178 ± 94 days vs. 178 ± 94 days), or duration of onset (92.30 ± 149.13 days vs. 110.86 ± 95.85 days). Significant differences were detected in gender (male/female: 22/24 vs. 35/7), family history (yes/no: 13/33 vs. 29/13), birth status (normal/abnormal: 30/16 vs. 30/16), metabolic status (normal/abnormal: 36/10 vs. 11/31), and brain MRI examination (normal/abnormal: 26/20 vs. 12/30) (Table 1).

**Table 1:** Comparisons between the etiology-specific group and non-etiology-specific group

	Etiology-specific group (n = 46)	Non-etiology-specific group (n = 42)	X^2^/t	*P*
Age (day)	255.93 ± 173.93	254.74 ± 119.03	-0.037	0.970
Gender (male/female)	22/24	35/7	12.131	**≤ 0.001**
Family history (yes/no)	13/33	29/13	14.639	**≤ 0.001**
Birth status (normal/abnormal)	30/16	9/33	17.059	**≤ 0.001**
Metabolic status (normal/abnormal)	36/10	11/31	23.922	**≤ 0.001**
brain magnetic resonance imaging (MRI) (normal/abnormal)	26/20	12/30	6.991	**0.008**
Age of onset (day)	178 ± 94	167 ± 98	-0.178	0.859
Duration of onset (day)	92.30 ± 149.13	110.86 ± 95.85	0.687	0.494

### Prediction of risk factors for non-etiology-specific infantile spasms

Logistic regression analysis showed that gender [odds ratios (OR) = 20.068, 95% (confidence interval) CI = 3.387–118.908, *P* = 0.001] and metabolic abnormalities (OR = 9.479, 95%CI = 1.935–46.449, *P* = 0.006), but not the birth status or brain MRI, were independent risk factors for IS in the non-etiology-specific group (Table 2).

**Table 2:** Analysis of risk factors of infantile spasms in children of the non-etiology-specific group

	B	S.E.	Wald	df	*P*	Odds ratios (OR)	95% confidence interval (CI)
Gender (male)	2.999	0.908	10.915	1	**0.001**	20.068	3.387-118.908
Family history	-0.866	0.863	1.007	1	0.316	0.421	0.078–2.282
Birth status abnormalities	-1.564	0.859	3.315	1	0.069	0.209	0.039–1.127
Metabolic abnormalities	-2.249	0.811	7.694	1	**0.006**	9.479	1.935–46.449
Abnormal brain magnetic resonance imaging (MRI)	0.153	0.979	0.025	1	0.876	1.166	0.171–7.942

### Baseline characteristic of children in the remission and nonremission groups

Seventy-seven children received adrenocorticotropic hormone (ACTH) treatment, and 84 children received antiepileptic drugs or anticonvulsant medications. Symptoms were relieved in 45 subjects (remission group) and unrelieved in 43 subjects (nonremission group). Clinical data were compared between the remission and nonremission groups. Family history (yes/no: 6/39 vs. 36/7), birth status (normal/ abnormal: 34/11 vs. 5/38), metabolic status (normal/ abnormal: 39/6 vs. 8/35), and brain MRI (normal/ abnormal: 33/12 vs. 5/38) were significantly different between the two groups. No significant differences in age (267.71 ± 169.50 vs. 242.44 ± 125.74), gender (male/female: 30/15 vs. 27/16), duration of onset (105.09 ± 151.25 vs. 97.05 ± 94.74), or time of therapy (28.07 ± 9.88 vs. 28.19 ± 10.78) were detected between the two groups (Table 3).

**Table 3:** Comparisons between unrelieved and relieved children after treatment

	Remission (n = 45)	Non-remission (n = 43)	χ^2^/t	*P*
Age (day)	267.71 ± 169.50	242.44 ± 125.74	-0.791	0.431
Gender (male/female)	30/15	27/16	0.145	0.704
Family history (yes/no)	6/39	36/7	43.667	**≤ 0.001**
Birth status (normal/ abnormal)	34/11	5/38	36.415	**≤ 0.001**
Metabolic status (normal/abnormal)	39/6	8/35	40.935	**≤ 0.001**
Brain magnetic resonance imaging (MRI) (normal/abnormal)	33/12	5/38	34.124	**≤ 0.001**
Age of onset (day)	183 ± 132	162 ± 127	-0.333	0.740
Duration of onset (day)	105.09 ± 151.25	97.05 ± 94.74	-0.297	0.767
Time of therapy (day)	28.07 ± 9.88	28.19 ± 10.78	0.054	0.957

### Prediction of risk factors for IS children unrelieved after treatment

Logistic regression analysis demonstrated that family history (OR = 0.114, 95%CI = 0.016–0.822, *P* = 0.031), birth status (OR = 0.039, 95%CI = 0.002–0.832, *P* = 0.038) and different etiologies (OR = 32.290, 95%CI = 1.137–917.223, *P* = 0.042), but not age, gender, or brain MRI, were independent risk factors for unrelieved symptoms after treatment (Table 4).

**Table 4:** Analysis of risk factors for unrelieved IS children after treatment

	B	S.E.	Wald	df	*p*	OR	95%CI
Family history	-2.167	1.006	4.645	1	**0.031**	0.114	0.016–0.822
Birth status	-3.253	1.566	4.316	1	**0.038**	0.039	0.002–0.832
Etiology grouping	3.475	1.707	4.141	1	**0.042**	32.290	1.137-917.223
Metabolic abnormalities	2.043	1.167	3.064	1	0.080	7.714	0.783–75.984
Abnormal brain magnetic resonance imaging (MRI)	-2.501	1.447	2.986	1	0.084	0.082	0.005–1.399

## Discussion

IS is more prevalent in male infants and is characterized by spasticity, electroencephalographic cerebral dysrhythmia, and etiology-specific delay. IS is caused by cranial nerve dysfunction in infants during the prenatal, perinatal, or postpartum period. IS can also be regarded as fetal epilepsy because the lesions usually occur in the embryo-fetal period (accounting for approximately 61%), and only a small group of lesions occur in the perinatal or postpartum period [16–18]. In agreement with previous studies, our results demonstrated that IS is more common in male children, and the main onset time of IS was 0–1 year old.

Significant differences in gender, family history, birth status, and brain MRI status were detected between the etiology-specific and non-etiology-specific groups. In a previous study exploring the clinical characteristics of 50 children with IS, 96% of the cases were etiology-specific IS and 4% were non-etiology-specific IS; intrapartum asphyxia was the leading cause of etiology-specific IS [19]. Gender may affect brain development and neonatal long-term neurological prognosis [20], which may explain why gender is a risk factor for non-etiology-specific IS in this study. Notably, MRI brain scans are recommended as the main neuroimaging modality to assess the etiology of IS [21]. Cortical structural abnormalities, mainly occurring in the frontal and temporal lobes, may be associated with non-etiology-specific IS [22]. However, in this study, brain MRI abnormalities were not risk factors for non-etiology-specific IS. This discrepancy may be due to the low number of non-etiology-specific infants in this study whose abnormal cortical structures mainly occurred in the frontal and temporal lobes.

Malformations of the cerebral cortex are usually associated with severe epilepsy and spasticity [23]. In this study, a high proportion of brain MRI abnormalities were observed in the non-etiology-specific and nonremission groups, including pachygyria, polymicrogyria, dysplastic corpus callosum, cortical dysplasia, and hemimegalencephaly. Verloes et al. [24] pointed out that brain malformations are the main cause of epileptic attacks in infants. In this study, brain MRI examination of IS patients revealed brain deformities; the findings of Howell et al. [25] were consistent with our results.

Additionally, the metabolic results of majority of the children in this study were not listed in the table because of the lack of metabolic diagnosis. However, two children exhibited a significant association of IS with inheritance, including one child with typical genetic epilepsy and one child with a TUBA1A 12q13.12, Exon4 heterozygous mutation. TUBA1A mutations may disrupt neuronal migration, are associated with brain malformations, and are characteristic of familial recurrence, indicating that TUBA1A mutations may result in IS [26, 27]. Furthermore, 25 innate metabolic errors resulting from single mutated genes cause heterogeneous conditions and may contribute to the etiology of IS [28]. Vitamin B12 deficiency results in severe neurological symptoms such as seizures and spasms, which gradually improve with vitamin B12 supplementation [29]. In this study, metabolic abnormalities were identified as a risk factor for non-etiology-specific IS.

Genetic testing, imaging, and other techniques can help determine the etiology of children with typical IS symptoms. In children with known IS etiologies, epilepsy can be controlled by inhibiting the negative feedback regulation produced by excessive corticotropin-releasing hormone secretion in the brain using adrenocorticotropic hormone (ACTH) [30, 31]. However, ACTH exhibits poor efficacy in children with non-etiology-specific IS, and these children are treated with anticonvulsant drugs [32]. In this study, 83 (83.38%) children were treated with ACTH in combination with anticonvulsants, such as sodium valproate and topiramate, and the symptom control rate was only 52.63% (50/95). Thus, the overall clinical efficacy was poor. Therefore, unless more etiologies of non-etiology-specific IS are discovered, clinical consideration should first be given to symptom relief, risk factor assessment, and preventive measures.

In agreement with a previous study, etiological grouping was a risk factor for unrelieved symptoms after treatment in children with IS [33]. Most children with etiology-specific IS received etiology-specific treatments. However, once neuronal damage occurs, the prognosis is poor even if clinical symptoms are controlled. Neuronal damage in children with non-etiology-specific IS may not appear before the onset but the efficacy and prognosis of established therapies for etiology-specific IS are often unsuitable for the treatment of non-etiology-specific IS. In this study, the age of IS onset was more than 3 months. After treatment, no significant difference in the age of onset between the remission and nonremission groups was detected, and the age of onset was not a risk factor for unrelieved symptoms after treatment.

A family history of epilepsy is associated with poor prognosis in epilepsy patients, while gender does not substantially affect the prognosis [34]. This is consistent with our study showing that family history, but not gender, is a risk factor for unrelieved IS symptoms after treatment. Perinatal neonatal hypoxia may also affect the occurrence of IS [35, 36]. In a clinical report of 30 cases of IS, premature infants accounted for about 26% of children with IS. In our study, asphyxia at birth increased the probability of drug treatment failure, which is consistent with the report by Gul et al. [33] showing that the long-term prognosis of IS is related to the etiology and family history.

There are several limitations to this study. Firstly, this paper was a single-center retrospective study with a relatively small sample size. Secondly, due to the limited data available, we could not establish the association of all risk factors with IS. Thus, further verification needs to be performed in large-scale, multicenter prospective studies.

## Conclusion

Giving attention to the gender and metabolic conditions of infants may help identify and prevent non-etiology-specific IS in infants. Additionally, a family history of epilepsy and the etiology of IS may suggest therapeutic effects of drugs. This study provides some reference values for the clinical treatment of IS.

## Data Availability

The data used to support the findings of this study are available from the corresponding author upon request.
